# Schiff Bases and Metal Complexes as Multifunctional Platforms: Bridging Bioinorganic Chemistry, Catalysis, Sensing, and Energy Applications

**DOI:** 10.1155/bca/8829417

**Published:** 2025-11-28

**Authors:** Luis A. Barreras-Contreras, Jonathan Moreno-Urbalejo, Iván F. Chávez-Urías, Damián F. Plascencia-Martínez, Diego Hernández-Martínez, Enrique F. Velázquez-Contreras, Karla-Alejandra López-Gastélum, Fernando Rocha-Alonzo

**Affiliations:** ^1^Department of Polymer and Materials Research, University of Sonora, Hermosillo, Sonora, Mexico; ^2^Department of Chemical Engineering and Metallurgy, University of Sonora, Hermosillo, Sonora, Mexico; ^3^Department of Food Research and Graduate Studies, University of Sonora, Hermosillo, Sonora, Mexico; ^4^Department of Chemical and Biological Sciences, University of Sonora, Hermosillo, Sonora, Mexico

**Keywords:** biological activity, catalytic activity, luminescent sensors, photovoltaic materials, Schiff base

## Abstract

Schiff bases are imine derivatives widely recognized for their structural versatility and ability to coordinate transition metals, giving rise to compounds with remarkable physicochemical and biological properties. Over the last decade, numerous studies have reported their diverse applications, ranging from antimicrobial, antioxidant, and anticancer activities to roles as catalysts, fluorescent sensors, and photovoltaic materials. While previous reviews have focused on specific aspects—such as biomedical activity, catalytic transformations, or luminescent sensing—there is still a lack of an integrative perspective that connects these different areas. In this review, we provide a comprehensive analysis of recent advances in Schiff bases and their metal complexes, emphasizing their multifunctionality at the interface of bioinorganic chemistry and materials science. We highlight how metal coordination enhances biological activity, how structural design expands the scope of asymmetric catalysis, how Schiff-based fluorophores are emerging as versatile luminescent sensors, and how aromatic and metal-Schiff derivatives contribute to the development of next-generation photovoltaic devices. By offering this transversal vision, the article aims to bridge fragmented knowledge and outline future research directions to fully exploit the potential of Schiff bases in medicine, catalysis, sensing, and sustainable energy.

## 1. Introduction

**Figure 1:**
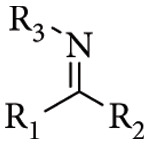
The specific structure fragment characteristic of Schiff bases, where R_1_, R_2_, and R_3_ are alkyl or (more often) aryl groups. R_1_ or/and R_2_ may also be hydrogen atoms.

The term Schiff base originates from the German chemist Hugo Schiff, who first reported in 1864 the products formed by the reaction between primary amines and carbonyl compounds. Schiff bases are classified as imine derivatives, characterized by the presence of a hydrocarbyl substituent attached to the nitrogen atom R_1_R_2_C = NR_3_ (R_3_ ≠ H) (Figure 1) [1]. These are condensation products of ketones or aldehydes with primary amines [2].

**Figure 2:**
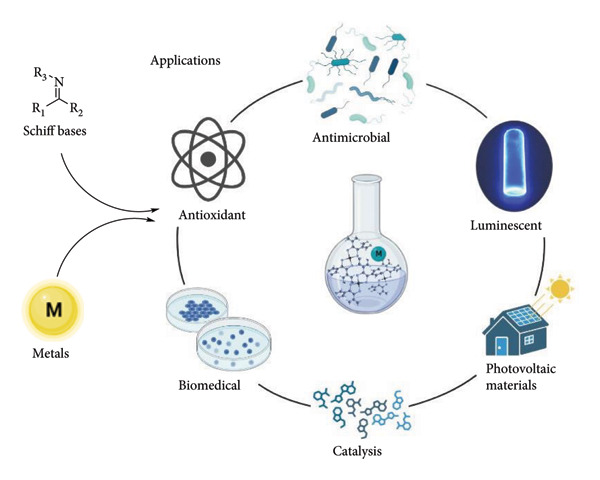
Main applications of Schiff bases. Crystal structure taken from [17].

Schiff bases are considered privileged ligands since they are relatively easy to synthesize and allow the introduction of stereogenic centers and other chirality elements in a simple way, so a wide variety of compounds can be obtained by selecting the appropriate starting material [3]. They also present a wide variety of applications in many fields such as the food industry [4], dye industry [5], catalysis [6], luminescence chemosensors [7], intermediates in organic synthesis [8], in material chemistry for applications in photoactive solar energy [9, 10], and in agrochemicals [11]. In biological chemistry [12], have demonstrated a broad range of biological activities, including antimalarial [13], analgesic, anti-inflammatory [14], antiviral, antifungal, antipyretic [15], and antibacterial [16] properties (Figure 2).

Ligand-based metal complexation has been extensively investigated for diverse applications, with particular emphasis on biological activity in the development of novel therapeutic agents. Schiff bases, in particular, can serve as versatile multifunctional ligands capable of coordinating with a wide variety of metal ions across different geometries and oxidation states. Both d-block transition metals and lanthanides readily form stable complexes with Schiff bases. Among these, sulfonamide ligand–based metal complexes have attracted considerable attention in pharmaceutical and biochemical research due to their broad spectrum of biological activities [18, 19].

In recent decades, these systems have demonstrated a remarkable spectrum of applications, ranging from biological activities (antimicrobial, antiviral, anticancer, and antioxidant) to the design of asymmetric catalysts, luminescent sensors, and optoelectronic materials. Several reviews have addressed specific aspects of this field, for instance, antimicrobial and pharmacological applications [12, 20], asymmetric catalysis and the development of metal complexes [21], or fluorescent probes for metal ion detection [22]. However, these contributions tend to focus on a single area of application.

In this context, a gap in the literature can be identified: to date, there is no integrative review encompassing the most relevant advances in Schiff bases and their metal complexes over the past decade, bridging biomedical, catalytic, and materials science applications. The present article seeks to fill this gap by providing a transversal perspective, highlighting how the structural and electronic properties of Schiff bases connect bioinorganic chemistry with the design of sensors and energy-related materials.

## 2. Antimicrobial, Antifungal, and Antiviral Activity

Schiff bases, organic compounds stemming from the condensation of aldehydes with primary amines, have emerged as pivotal subjects of scientific inquiry due to their multifaceted bioactive characteristics [23]. Their intrinsic structural features render them intriguing candidates for exploration as antimicrobial, antifungal, and antiviral agents. The versatility of Schiff bases lies in their molecular architecture, which enables interactions with diverse biological targets crucial for pathogen inhibition. Their antimicrobial prowess extends to impeding the proliferation and viability of a broad spectrum of microbial pathogens, encompassing bacteria, fungi, and viruses [24]. For clarity in the discussion that follows, we use standard microbiological terms as follows: minimum inhibitory concentration (MIC), the lowest concentration that prevents visible growth; minimum bactericidal concentration (MBC), the lowest concentration that kills ≥ 99.9% of the initial inoculum; inhibition zone, the diameter of the clear halo observed in agar diffusion assays; and the MTT assay, a colorimetric readout of cellular metabolic activity used to estimate viability. This unique attribute underscores the potential utility of Schiff bases as therapeutic modalities in combating infectious diseases, both conventional and emerging. Moreover, the intricate interplay between their chemical structure and biological activity incites endeavors toward synthesizing novel derivatives and refining existing frameworks to enhance their pharmacological efficacy and target specificity [25]. Through an in-depth examination, this review aims to elucidate the structural-functional relationships underpinning the antimicrobial, antifungal, and antiviral activities of Schiff bases, elucidating their implications and prospects across medical and pharmaceutical domains.

**Figure 3:**
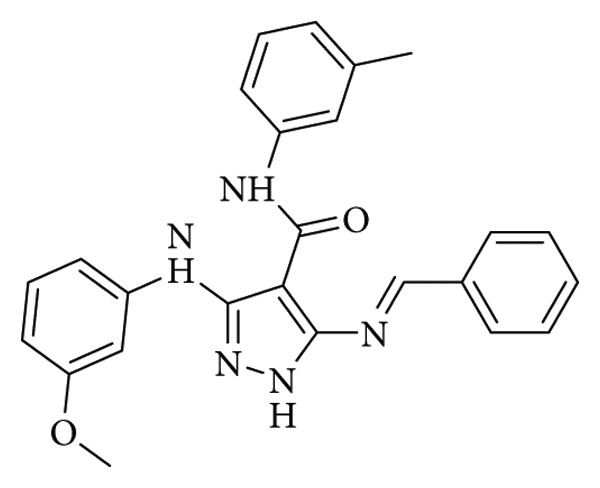
Compound 18 (5-aminopyrazole–derived Schiff base) reported by Hassan et al. [26].

In their 2019 study, Hassan et al. examined the antimicrobial potential of 5-aminopyrazole–derived compounds. These compounds demonstrated remarkable activity against multidrug-resistant bacteria. Schiff base 18 (Figure 3) showed very good activity against *Staphylococcus aureus* (MIC: 15.62 μg/mL). Further enzymatic assay aided by molecular docking study demonstrated that compound 18 is a potent inhibitor of *S. aureus* DNA gyrase and dihydrofolate reductase kinases. This study could be valuable in the discovery of new potent antimicrobial agents [26].

**Figure 4:**
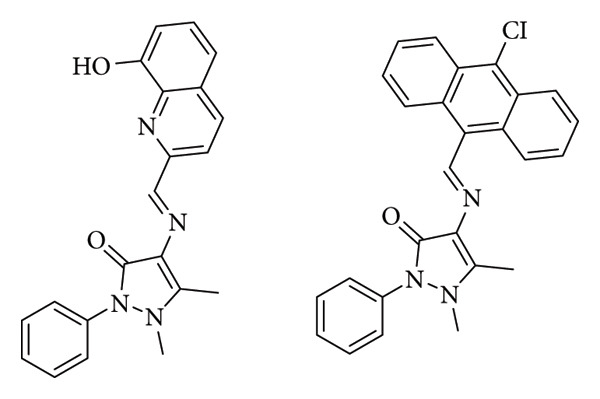
Compounds 3a (4-(((8-hydroxyquinolin-2-yl)methylene)amino)-1,5-dimethyl-2-phenyl-1,2-dihydro-3H-pyrazol-3-one) and 3b (4-(((10-chloroanthracen-9-yl)methylene)amino)-1,5-dimethyl-2-phenyl-1,2-dihydro-3H-pyrazol-3-one) [27].

Erturk synthesized two imine derivatives (compounds 3a and 3b) (Figure 4). These compounds were evaluated for their antimicrobial and antioxidant properties. According to MIC results, compounds 3a and 3b showed a high antifungal activity against the fungus *Aspergillus niger* (MIC: 25 μg/mL and 12.5 μg/mL, respectively) compared to the activity observed on nystatin (12.5 μg/mL). The compounds were found to exhibit more potent inhibitory activity on Gram-positive bacteria. Both compounds showed a high activity against Gram-positive bacteria *Micrococcus luteus* and *S. aureus* (MIC = 25 μg/mL and 12.5 μg/mL, respectively, compared to ampicillin's MIC = 100 μg/mL and 12.5 μg/mL) [27].

**Figure 5:**
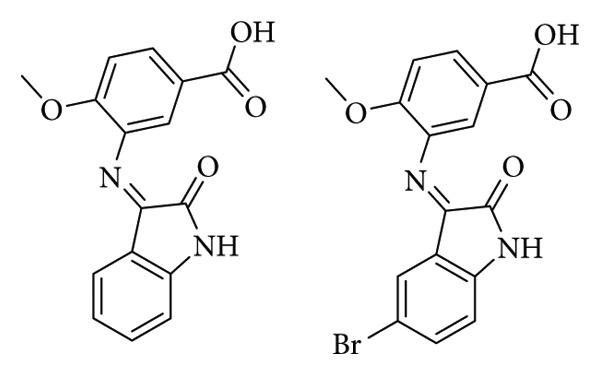
Compound 2: (E)-2-isopropyl-N-(2-nitrobenzylidene)aniline [28].

Yusuf et al. synthesized and assessed the antimicrobial activity of three Schiff base derivatives testing them against *Escherichia coli*, *Salmonella typhimurium*, and *Pseudomonas Aeruginosa*. Compound 2 (Figure 5) exhibited strong inhibition against Gram-negative bacteria *S. typhimurium* and *P. aeruginosa* (MIC = 15.625 μg/mL and 7.81 μg/mL), outperforming chloramphenicol (MIC = 31.25 μg/mL and 62.50 μg/mL). By contrast, compounds 1 and 3 showed only moderate activity against *S. typhimurium* and no activity against *P. aeruginosa*, and none of the three derivatives surpassed chloramphenicol against *E. coli*; all three were inactive against *S. aureus*. A molecular docking analysis was conducted to validate the antibacterial effects of the compounds. Results indicated that compound 2 displayed stronger inhibitory activity against the target enzymes compared to the other compounds, which was supported by the observed formation of hydrogen bonds [28].

**Figure 6:**
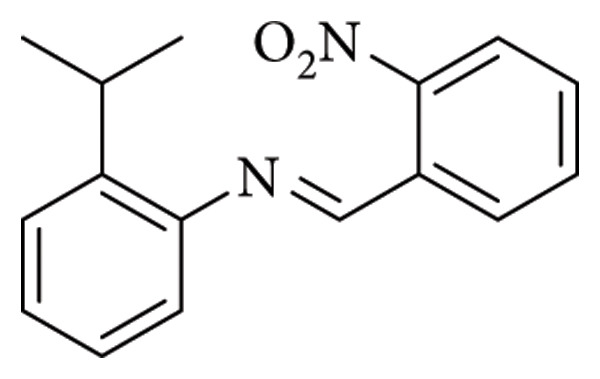
Compounds 1a (4-methoxy-3-(2-oxo-1,2-dihydro-indol-3-ylideneamino)benzoic acid) and 1b (3-(5-bromo-2-oxo-1,2-dihydro-indol-3-ylideneamino)-4-methoxybenzoic acid) [29].

Chemchem et al. conducted research on twelve isatin Schiff bases, focusing on their antimicrobial activity. The compounds were evaluated against standard and clinical bacterial strains via agar-well diffusion. Compounds 1a and 1b (Figure 6) proved to have the lowest MIC (78 mg/mL) against *P*. *aeruginosa* with a 24 mm an 15 mm inhibition zone, respectively, while the antibiotic fosfomycin showed a zone of 16 mm for 200 mg/mL [29].

**Figure 7:**
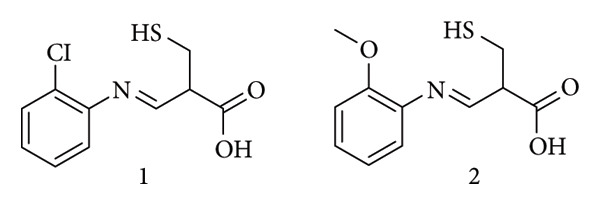
Compounds 1 (2-((2-chlorobenzylidene)amino)-3-mercaptopropanoic acid) and 2 (3-mercapto-2-((2-methoxybenzylidene)amino)propanoic acid) [30].

Salihović et al. outlined the synthesis of two imine derivatives (Figure 7). Their work encompassed a comprehensive evaluation of antimicrobial activity against multiple Gram-positive and Gram-negative bacterial strains (*E. coli, Salmonella enterica, Proteus hauseri, P*. *aeruginosa, Klebsiella pneumoniae, S*. *aureus, Bacillus subtilis, M*. *luteus, M. flavus,* and *Clostridium sporogenes*). Compound 1 demonstrated potent antimicrobial efficacy against all bacterial strains (MIC = 1.284 mM), while compound 2 displayed relatively lower activity (MIC = 2.612 mM). Additionally, both compounds exhibited notable antifungal activity against *Aspergillus brasiliensis* (MIC = 1.284 mM and 1.306 mM, respectively). It may be inferred that compound 1 exhibits enhanced activity owing to the presence of a chlorine substituent, which significantly increases the molecule's lipophilicity and, consequently, its biological activity [30].

**Figure 8:**
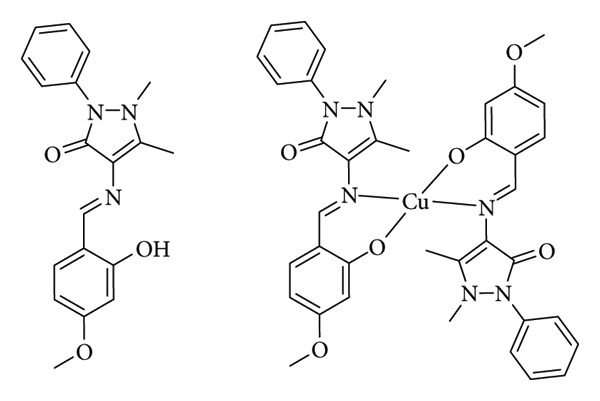
HL3 ligand and complex C3 (bis-N,O-bidentate Schiff-base Cu(II) complex) reported by Kargar et al. [31].

Kargar et al. synthesized novel Cu(II) complexes with bis-N,O-bidentate Schiff base ligands (Figure 8), investigating substitution effects on antimicrobial activity. Compared to the ligand (HL3), the complex (compound C3) exhibited enhanced bactericidal and inhibitory effects in a limited range, with MBC/MIC values of 128/64 μg/mL against *E. coli* and 64/32 μg/mL against *S. aureus*. This enhancement was attributed to coordination bond formation, following overtone's concept and Tweedy's chelation theory. Coordination reduced the central metal ion's polarity, facilitating interaction with sensitive cellular membranes due to decreased positive charges and increased atomic radius. Furthermore, π-electron delocalization from coordination increased complex lipophilicity, promoting efficient penetration of microorganism lipid layers for enhanced eradication [31].

**Figure 9:**
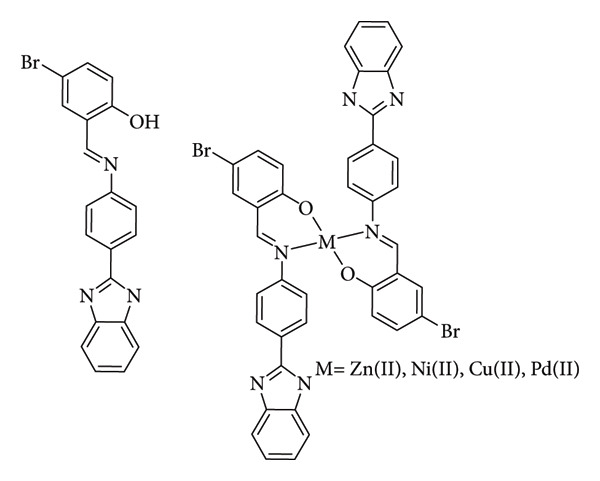
Compound 1: (E)-2-((4-(1H-benzo[d]imidazol-2-yl)phenylimino)methyl)-4-bromophenol, and its Zn(II), Ni(II), Cu(II) and Pd(II) complexes [32].

Mahmood et al. investigated a benzimidazole Schiff base derivative along with its corresponding Zn(II), Ni(II), Cu(II), and Pd(II) complexes (Figure 9). The synthesized ligand and complexes were assessed for antibacterial activity, the complexes presented better activity than the ligand, and particularly the Ni(II) complex demonstrated the most favorable antibacterial outcomes, which correlated with its strong binding affinity to DNA. Additionally, the Zn(II) complex exhibited notable antibacterial efficacy against *M. luteus, E. coli*, and *Enterobacter aerogenes*, in line with its observed DNA binding capabilities. Although the Cu(II) complex displayed promising antibacterial activity specifically against *M. luteus*, its effectiveness did not coincide with its relatively poor DNA binding. Previous studies have suggested that the antibacterial effects of Cu(II) complexes might stem from their ability to damage bacterial membranes and inhibit the expression of certain extracellular proteins [32].

**Figure 10:**
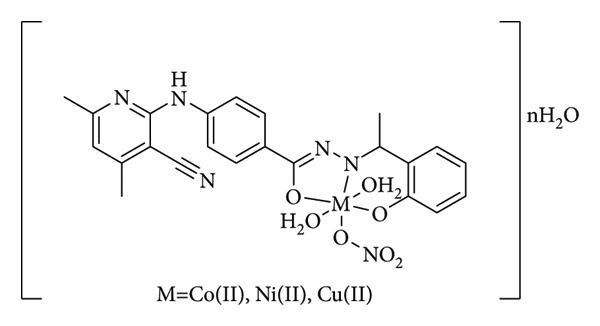
Schiff base from 2-hydroxyacetophenone and 4-(3-cyano-4,6-dimethylpyridin-2-ylamino)benzohydrazide, and its Co(II), Ni(II) and Cu(II) complexes [33].

El-Gammal et al. presented a novel Schiff base compound synthesized through the condensation of 2-hydroxyacetophenone with 4-(3-cyano-4,6-dimethylpyridin-2-ylamino)benzohydrazide. This compound, along with its Co(II), Ni(II), and Cu(II) complexes (Figure 10), exhibited notable antibacterial activity against *S*. *aureus*, with inhibition zones of 12, 12, 11, and 10 mm for the ligand (HL), complex (1), complex (2), and complex (3), respectively. On the other hand, all the compounds showed no antifungal activity against *Candida albicans* except HL [33].

**Figure 11:**
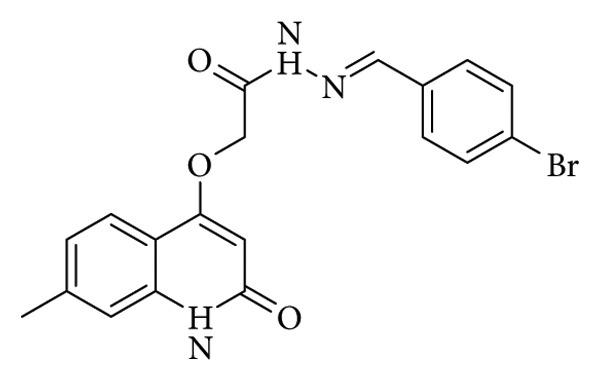
Chemical structure of compound 4b, (E)-N′-(4-bromobenzylidene)-2-((7-methyl-2-oxo-1,2-dihydroquinolin-4-yl)oxy)acetohydrazide, as reported by Alshammari et al. [34].

Alshammari et al. studied a series of Schiff base derivatives. Molecular docking studies demonstrated that the tested compounds displayed strong binding affinity toward the main protease (M^pro^), comparable to remdesivir. Analysis of the docked complex of (E)-N'-(3-methoxybenzylidene)-2-((7-methyl-2-oxo-1,2-dihydroquinolin-4-yl)oxy)acetohydrazide with RdRp (Figure 11) revealed the establishment of three key hydrogen bonds with TYR619, ASP623, and GLU811 residues, exhibiting average bond lengths of 2.19, 2.12, and 2.24 Å, respectively. Collectively, these findings suggest that the synthesized compounds may serve as promising inhibitors of SARS-CoV-2 M^pro^ [34].

**Figure 12:**
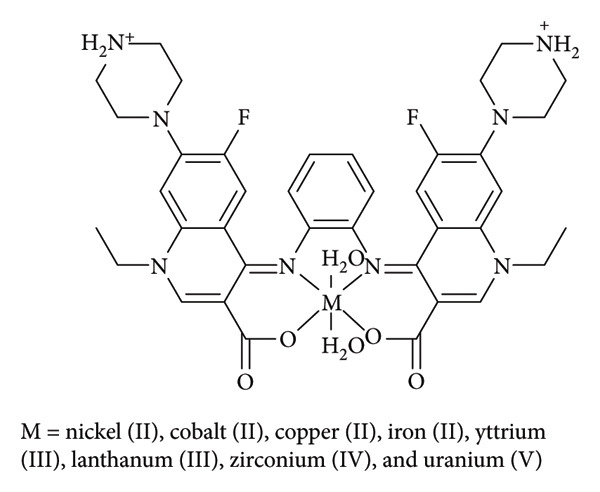
Structures of Ni(II), Co(II), Cu(II), Fe(III), Y(III), and La(III) complexes derived from the Schiff base ligand Nor-o-phdn·2H_2_O, as reported by Abd El-Hammid et al. [35].

Abd El-Hammid et al. presented a new Schiff base ligand and its nickel(II), cobalt(II), copper(II), iron(III), yttrium(III), lanthanum(III), zirconium(IV), and uranium(VI) complexes (Figure 12). The compounds were evaluated by their antibacterial, antifungal, and antiviral activity. The in vitro antibacterial bioactivity of the metal complexes demonstrated potent inhibitory effects against *E. coli* and *S. typhi* strains compared to the parent ligand. Metal complexes exhibited superior activity against *S. aureus*. Specifically, Ni(II) and Co(II) complexes displayed significant antibacterial effects against *Bacillus cereus*, suggesting their potential for new antibiotic development. However, no substantial antifungal bioactivity was observed against *A. niger* and *Penicillium vulpinum*. Notably, the anti-huCMV (human cytomegalovirus) effect of the synthetic compounds was evaluated in humanized-infected mice, revealing that the Schiff base lacked antiviral bioactivity. Conversely, complexation with Fe(III) and Zr(IV) metals effectively reduced huCMV replication, indicating potential for antiviral treatment development. Nonetheless, further large-scale clinical studies are warranted [35].

**Figure 13:**
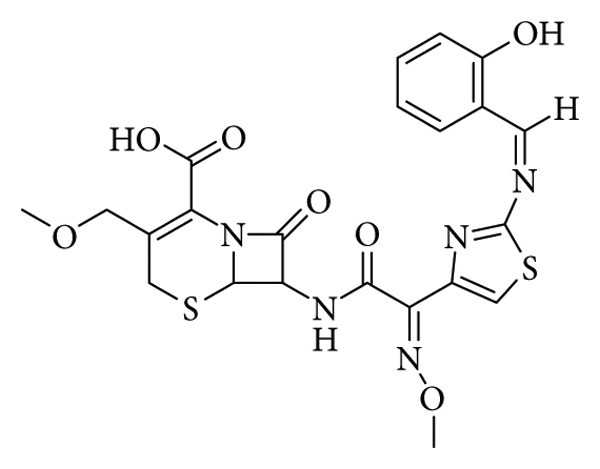
7-((E)-2-(2-((Z)-(2-hydroxybenzylidene)amino)thiazol-4-yl)-2-(methoxyimino)acetamido)-3-(methoxymethyl)-8-oxo-5-thia-1-azabicyclo[4.2.0]oct-2-ene-2-carboxylic acid (cefpodoxime-Schiff-base derivative) [36].

Mahmood et al. presented some cefpodoxime Schiff base derivatives (Figure 13), in this work, was found in antibacterial assays against *Bacillus subtilis*, Schiff base C1 demonstrated moderate activity (3mm inhibition zone) compared with the parent drug, cefpodoxime. However, a notable increase in efficacy was observed against *Stenotrophomonas maltophilia*, *Serratia marcescens*, and *Escherichia coli* (30, 50, and 25 mm inhibition zone, respectively), outperforming cefpodoxime. C1 also showed highly strong antiviral potential against avian influenza (H9) and avian corona (IBV) viruses (half-maximal inhibitory concentration [IC_50_] = 16 and IC_50_ = 64, respectively). In elucidating the molecular interactions underlying the observed biological activities, computational analyses revealed crucial binding sites within the thumb, finger, and palm domains of the IBV PLPro enzyme. Schiff base C1 displayed a notable binding energy of −7.8 kcal/mol, indicative of favorable interactions within the enzyme's active site [36].

## 3. Antioxidant

The antioxidant capacity of Schiff bases has sparked great interest in the scientific community. These molecules, both in their free form and as metal complexes, have proven to be effective in protecting against oxidative stress [37]. Antioxidants play a crucial role in the oxidant/antioxidant balance by preventing or reducing the excessive production of free radicals. While they are necessary for proper cellular function at low concentrations, an excess of free radicals can cause oxidative damage to various biomolecules present in the cellular environment, including proteins, lipids, and DNA [38–41].

Numerous studies have been conducted to evaluate the antioxidant activity of Schiff bases in different compounds, yielding promising results. These molecules have demonstrated their ability to neutralize free radicals and protect biomolecules from oxidative damage [42, 43]. Here are some notable examples of these antioxidant assays.

**Figure 14:**
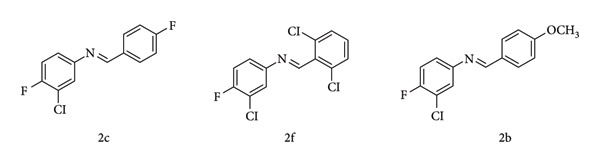
Examples of Schiff bases synthesized by Mermer et al. from 4-methylaniline and 3-chloro-4-fluoroaniline [44].

One example is the study by Mermer et al. in 2018, where they synthesized 18 aromatic imine compounds from 4-methylaniline and 3-chloro-4-fluoroaniline (Figure 14). The tests were based on a spectrophotometric principle that resulted in stable color radical reduction. These compounds were assessed for their antioxidant activity using 2,2-diphenyl-1-picrylhydrazyl (DPPH), cupric reducing antioxidant capacity (CUPRAC), and ferric reducing antioxidant power (FRAP) assays [44].

The results revealed that Schiff bases derived from 3-chloro-4-fluoroaniline exhibited the highest antioxidant activity. In Table 1, the outcomes of all the compounds are presented, with special emphasis on compounds 2c, 2f, and 2b, which demonstrated moderate to good activities with IC_50_ values of 0.15 ± 0.01, 0.19 ± 0.01, and 0.22 ± 0.01 mM/mL, respectively, in the DPPH assay, compared to the Trolox standard (IC_50_ = 0.04 ± 0.01). Moreover, these compounds also displayed significant antioxidant activities in the FRAP and CUPRAC assays, with values of 4490 ± 20, 4230 ± 10, and 4150 ± 10 (for FRAP) and 8890 ± 10, 8880 ± 20, and 8160 ± 20 (for CUPRAC), respectively [44].

**Figure 15:**
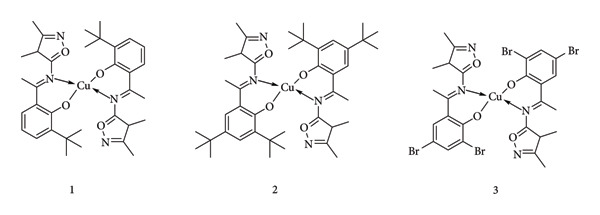
Copper(II) complexes with isoxazole-derived Schiff-base ligands [45].

Schiff bases are well-known compounds for the formation of metal complexes, especially with transition metals, which have also been tested in antioxidant assays. In 2018, Nirmala Ganji and colleagues conducted an antioxidant assay using copper(II) complexes with Schiff bases as ligands (Figure 15). The assays were based on a spectrophotometric principle that resulted in the reduction of stable-colored radicals. The scavenging activity of the prepared compounds was examined by measuring the decrease in absorbance at 517 nm corresponding to the DPPH free radical. The IC_50_ was calculated and compared with ascorbic acid (AA) (a known antioxidant). The results (Table 2) showed that complex 3 exhibited higher antioxidant activity (0.42 mM (AA), 7.84 mM (1), 9.82 mM (2), and 3.57 mM (3)) [45].

These findings highlight the remarkable antioxidant potential of Schiff bases, both in their free form and as metal complexes. They play a crucial role in maintaining the oxidant/antioxidant balance by preventing or reducing excessive production of free radicals. As such, Schiff bases have emerged as promising candidates for protecting biomolecules from oxidative damage, including proteins, lipids, and DNA [46].

## 4. Anticancer

Schiff bases have shown a potential application as anticancer agents. At present, cancer remains the foremost cause of mortality across the globe, and although multiple therapeutic strategies are employed, chemotherapy continues to be regarded as one of the most effective approaches. Therefore, the development of new compounds with antitumor properties is of great importance in the scientific community. Several research studies have demonstrated the potential of Schiff bases as inhibitors of cancer cells [47–50]. Below, some notable examples are mentioned.

**Figure 16:**
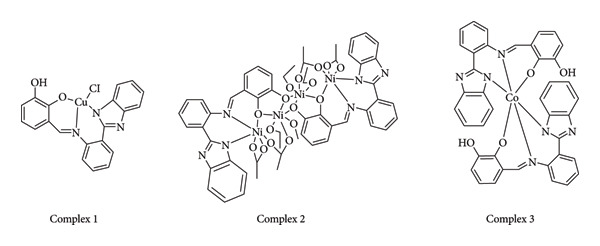
Metal complexes with Schiff base ligands developed by Hou et al. [51].

Min Hou and colleagues in 2023 developed various Cu(II), Ni(II), and Co(II) complexes using Schiff base ligands derived from 2,3-dihydroxybenzaldehyde-2-(2-aminophenyl)benzimidazole (Figure 16). The antitumor activity of the three complexes was determined using the MTT method on human cell lines MAD-MB-231, A549, CNE-2Z, and SMMC-7721. The results are shown in Table 3, where it can be observed that complexes 1–3 exhibit significant anticancer activity, with complex 2 showing the strongest activity against all four human cell lines. Complex 2 also demonstrated lower cytotoxicity than the standard drug cisplatin (a known anticancer medication) in CNE-2Z cells [51].

**Figure 17:**
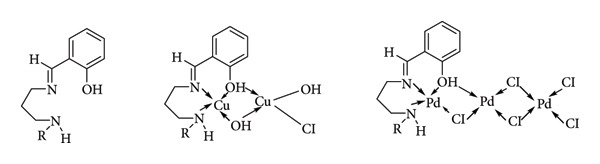
Examples of Schiff base ligands (HL^3^) and their copper and palladium complexes synthesized by Emam and colleagues [52].

Emam and colleagues achieved the synthesis of novel Schiff bases derived from neocryptolepine, along with four metal complexes using copper(II), cobalt(II), nickel(II), and palladium(II) (Figure 17). Both the ligands and their corresponding metal complexes were subjected to an anticancer activity evaluation against colon carcinoma cell line (HT-29) using the MTT assay. The cell inhibition percentage was measured after 72 h for each compound, and the results are presented in Table 4. Remarkably, some compounds exhibited high cellular inhibition (up to 87%), particularly those derived from the third ligand (HL^3^) [52].

In summary, Schiff bases constitute a class of chemical compounds that have displayed significant potential in anticancer therapy, both as ligands and in the form of metal complexes [53, 54]. Their antitumoral activity has been ascribed to diverse mechanisms, and ongoing research aims to further explore their potential as therapeutic agents in the battle against cancer [50, 55].

## 5. Catalytic Activity

The Schiff base ligands exhibit noteworthy versatility in their ability to form complexes with a diverse range of metals. While copper, rhodium, ruthenium, and zinc are commonly utilized, other metals such iridium, lanthanides, and samarium, among others [56–58], also demonstrate compatibility with these ligands. These metal complexes have demonstrated remarkable catalytic activity in the enantioselective synthesis of alcohols, amines, nitriles, and cyclopropanes [59–61]. These catalysts have shown enhanced efficiency and selectivity, making them highly valuable in asymmetric transformations.

**Figure 18:**
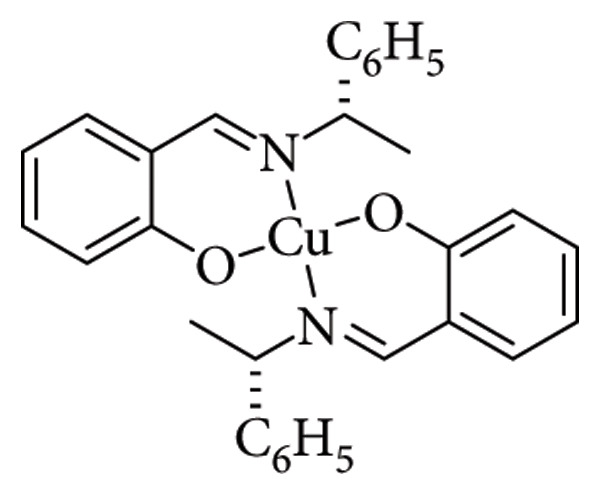
Nozaki's catalyst [62].

The initial report of a transition metal complex using a Schiff base ligand capable of producing enantiomeric excess in a reaction dates back to the 1960s. Nozaki pioneered this discovery by employing a copper(II) complex (Figure 18) to synthesize aromatic *cis/trans* cyclopropanecarboxylates from styrene and ethyl diazoacetate, achieving enantiomeric excesses of 10% and 6%, respectively [62].

**Figure 19:**
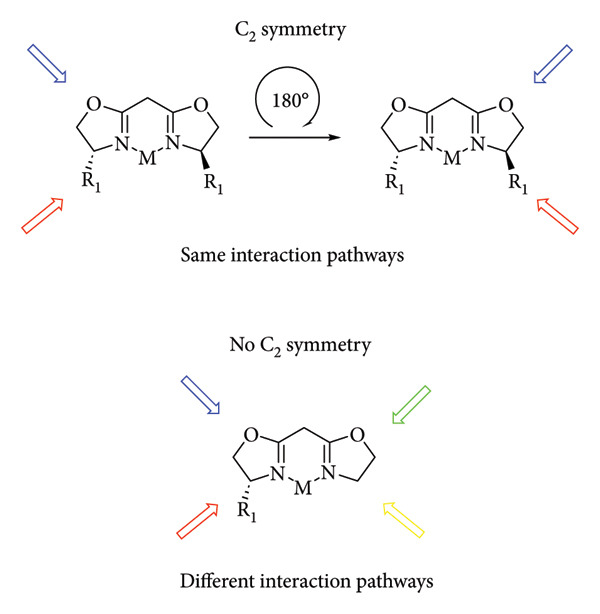
C_2_ symmetry complex interaction with substrate [64].

Copper, rhodium, ruthenium, and palladium complexes allow a greater stereocontrol in the reaction [63]. A desirable feature in ligands used for enantioselective synthesis is symmetry, with C_2_ symmetry being the most common. Although the exact reason why C_2_-symmetric ligands enhance stereocontrol is not fully understood, they restrict the ways in which the catalyst can interact with the substrate. This limitation reduces the number of distinct catalyst–substrate complexes and, consequently, the variety of products [64]. As illustrated in Figure 19, in complexes with C_2_-symmetric ligands, the presence of two equivalent substituents (indicated by the blue and red arrows) creates an identical coordination environment even upon a 180° rotation. In contrast, asymmetric ligands have nonequivalent substituents, shown by arrows of different colors, which give rise to multiple distinct coordination environments and a broader range of possible interactions.

Noyori was a pioneer in the synthesis of chiral alcohols through the hydrogenation of prochiral ketones using BINAP/diamine-ruthenium catalysts [65]. The use of gaseous hydrogen led to the development of techniques that utilize alternative hydrogen donors, such as 2-propanol, instead of gaseous hydrogen and ruthenium complexes [66]. In recent years, methodologies have been developed that eliminate the use of gaseous hydrogen and ruthenium, aiming to utilize alternative hydrogen sources and metals that are less toxic or more cost-effective than ruthenium. These methodologies involve the use of Schiff base ligand complexes with metals such as palladium, cobalt, and nickel [67–69].

**Figure 20:**
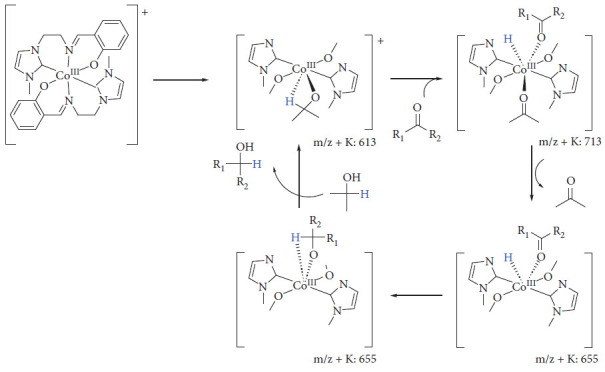
Abubakar and Bala hydrogenation [71].

Most of the proposed reaction mechanisms for hydrogenation involve the presence of an 18-electron precatalyst that is converted into a 16-electron hydride, which is the species that carries out the catalytic cycle [70].  Figure 20 shows the mechanism proposed by Abubakar using a Cobalt(III) complex [71].

**Figure 21:**
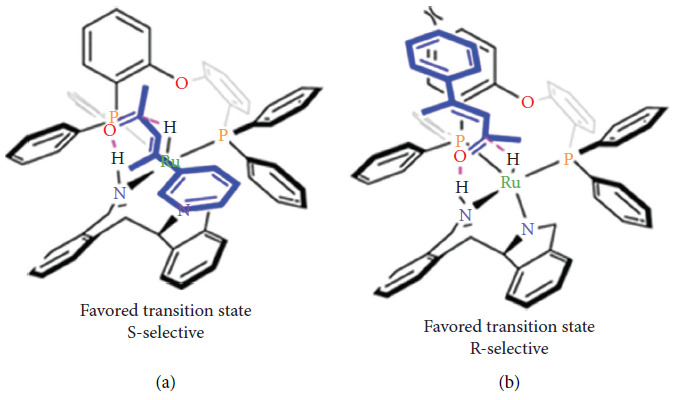
Different transition states induced by chiral ligands [72].

Furthermore, the previously mentioned C_2_ symmetry, the chirality of the ligand, is another aspect to take into account. To favor the formation of one of the enantiomers, it is necessary to block one of the enantiotopic faces of the substrate. This will promote the formation of the transition state that leads to the formation of the *R* enantiomer if the *Si* face is blocked, or the formation of the *S* enantiomer if the Re face is blocked [72]. In Figure 21, Chen illustrates this phenomenon with the hydrogenation of an enone using a diamine-Ru catalyst.

**Figure 22:**
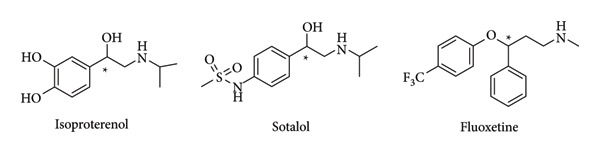
Structure of different drugs derived from a prochiral ketone [74].

There are many molecules, particularly drugs, directly derived from prochiral ketones such as isoproterenol, sotalol, fluoxetine [73, 74] (Figure 22), and others like levofloxacin, which require an asymmetric transfer hydrogenation reaction in their synthesis pathway. The development of catalysts for such reactions will facilitate the production of these important molecules.

**Figure 23:**
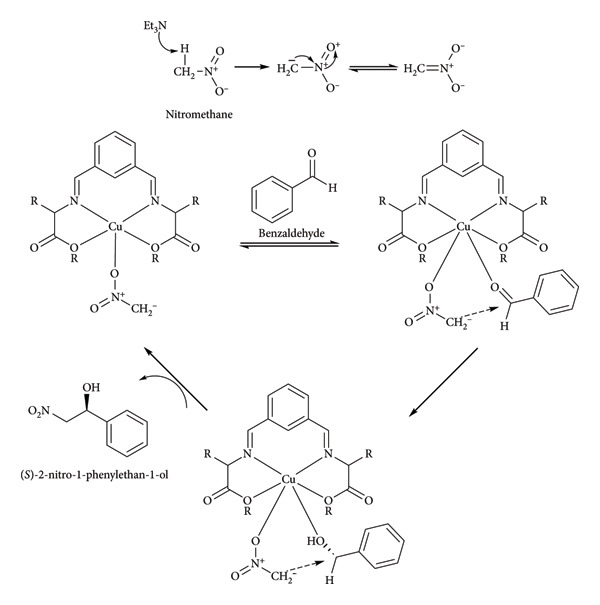
Kumar et al.'s Henry reaction [75].

An alternative in the use of prochiral ketones is the utilization of aldehydes, both aromatic and aliphatic, in the Henry reaction to generate β-nitro alcohols. The mechanism of the Henry reaction catalyzed by a complex with a Schiff base ligand was explained by Kumar in 2021. In the example, a C_2_-symmetric bisimine ligand coordinates a copper atom, nitromethane and triethylamine are required to deprotonate it, forming the nitronate ion, which coordinates to the metal center along with benzaldehyde and attacks its carbonyl group. Due to the spatial arrangement of the atoms, the ion can only attack the Re face of the carbonyl, resulting in a higher amount of the *S* enantiomer of 2-nitro-1-phenylethanol generated (Figure 23) [75].

**Figure 24:**
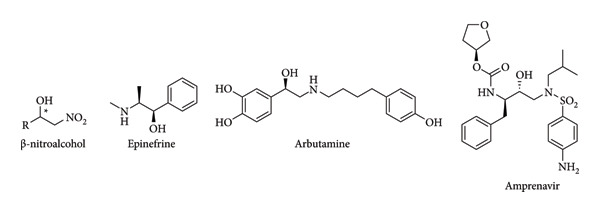
Some β-nitroalcohol derivatives [77, 78].

Important drugs such as amprenavir (HIV protease inhibitor) [76], arbutamine (coronary disease) [77], or epinephrine (treatment of anaphylaxis) [78] are derived from nitroalcohols and can be obtained in enantiomerically pure form by incorporating these types of reactions into their synthesis pathways (Figure 24).

**Figure 25:**
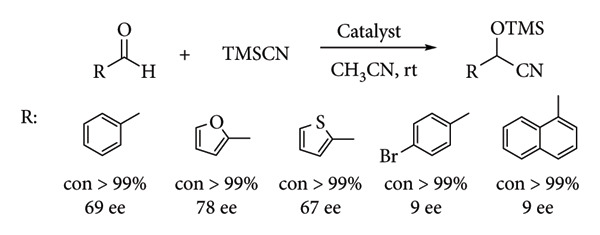
Weng et al.'s cyanosilylation [79].

A poor explored reaction using metal complexes with Schiff base ligands is the cyanosilylation of aldehydes. Weng et al. synthesized a Schiff base-type complex derived from o-vanillin and cobalt. The cyanosilylation reaction achieved high conversion and enantioselectivity (*ee*) (Figure 25) [79].

## 6. Luminescent Sensors

Luminescent sensors based on Schiff bases are widely used in analytical applications due to their sensitivity and selectivity for detecting certain analytes. Here are some examples of luminescent sensors using Schiff bases.

pH sensors. Schiff compounds can change their structure and luminescent properties depending on the pH of the environment. A pH sensor based on Schiff's bases could change color or emit fluorescent light at different wavelengths depending on the pH of the solution. These sensors are useful for monitoring pH changes in biological or environmental solutions. The development of fluorescent sensors targeted toward pH is crucial across various fields due to their ability to detect changes in the acidic-basic environment. These sensors provide a fundamental tool in biochemical, pharmaceutical, and environmental research, enabling the monitoring of biological processes, assessing drug efficacy, and controlling water quality, among other applications [80]. Fluorescence is highly sensitive to pH variations, making these sensors a versatile and effective option for pH detection and quantification in different mediums. The ability to design and optimize these sensors to specifically respond to pH changes within relevant ranges for biomedical and environmental applications is a significant advantage. Furthermore, their sensitivity and selectivity can be fine-tuned through molecular structure modifications, rendering them adaptable and versatile tools for a wide array of real-time pH detection applications [81].

**Figure 26:**
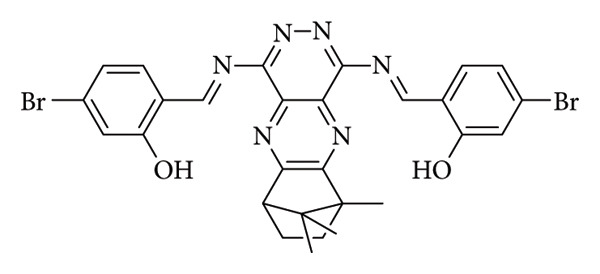
Camphor Schiff base pH probe reported by Tian et al. [82].

Tian et al. developed a fluorescent probe using camphor as a base (Figure 26). Camphor, a monoterpenoid present in Lauraceae plants, possesses a rigid double ring structure that limits intermolecular interactions and energy loss due to collisions. This unique structure makes it an ideal candidate for a fluorescent probe. The probe exhibited remarkable specificity in detecting pH changes in alkaline environments. It remained unaffected by various cations, anions, and amino acids. Additionally, it showcased swift responsiveness, reversibility, and a substantial Stokes shift. The fluorescence intensity at 491 nm was plotted against pH values (9.65–11.68), showing a strong correlation from which a pKa of 10.62 was determined using the Henderson–Hasselbalch equation. Upon investigating the detection mechanism, findings indicated that the phenolic hydroxyl group could deprotonate under alkaline conditions, potentially leading to intramolecular charge transfer (ICT) with the pyridazine ring of the probe. Furthermore, the probe's viability in real-world scenarios was explored. Due to the biocompatibility and cell permeability conferred by the camphor component, tests were conducted to assess its effectiveness in monitoring alkaline pH changes in living organisms like zebrafish, yielding positive outcomes [82].

**Figure 27:**
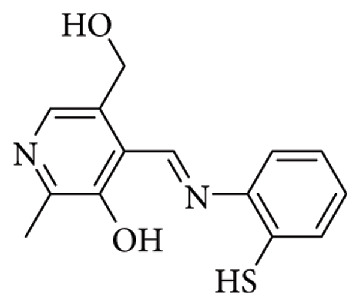
Pyridoxal Schiff base pH probe reported by Bamnavat et al. [83].

**Figure 28:**
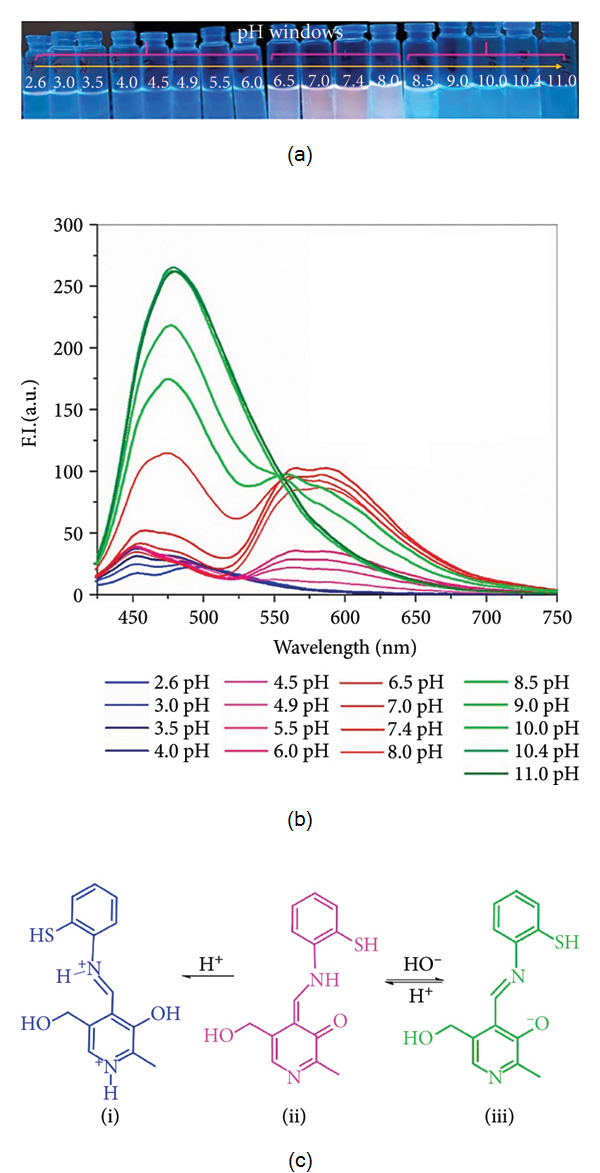
Graphical abstract of Bamnavat et al.'s work, showing (a) the vials irradiated with UV light at 365 nm, (b) the fluorescence spectra of the Schiff base in DMSO/HEPES mixture, and (c) the proposed mechanism for the pH dependent fluorescence switching of the Schiff base: (i) acidic, (ii) aggregation, and (iii) basic conditions [83].

Bamnavat et al. reported the synthesis of a Schiff base derived from pyridoxal, which was examined for its fluorescent pH sensing properties (Figure 27). Pyridoxal, a form of vitamin B6, is an organic molecule containing a pyridine ring with an aldehyde substituent, a structure that endows it with distinctive reactivity and makes it suitable for sensor applications. Its sensitivity to environmental changes such as pH fluctuations or the presence of metal ions renders pyridoxal-based compounds attractive platforms for fluorescence detection. In DMSO, the Bamnavat Schiff base displayed weak emission; however, upon increasing the water fraction in DMSO, the system became strongly red emissive. This enhancement was attributed to the combined effects of aggregation-induced emission (AIE) and excited-state intramolecular proton transfer (ESIPT). The ESIPT process in Schiff bases occurs through rapid conversion from the enol ground state to the keto form in the excited state. While intramolecular hydrogen bonding and C=N isomerization can suppress ESIPT in solution, aggregation restricts intramolecular rotation, thereby activating ESIPT and producing marked fluorescence enhancement. The resulting red emission band at 580 nm, with a large Stokes shift of approximately 220 nm, confirmed the synergistic role of AIE and ESIPT. For practical application, the self-aggregates of the Schiff base in a mixed DMSO:HEPES system (95% HEPES buffer, where HEPES = 4-(2-hydroxyethyl)-1-piperazineethanesulfonic acid) were used as a fluorescent pH sensor. At physiological pH, the Schiff base aggregates were red-emissive, but they shifted to green emission upon deprotonation of the pyridoxal–OH group. Analysis of fluorescence titration data across varying pH values yielded a pKa of 9.08 for the pyridoxal–OH moiety. Overall, the sensor exhibited three distinct fluorescence responses depending on pH: blue emission under acidic conditions, red emission at physiological pH, and green emission in basic media (Figure 28) [83].

**Figure 29:**
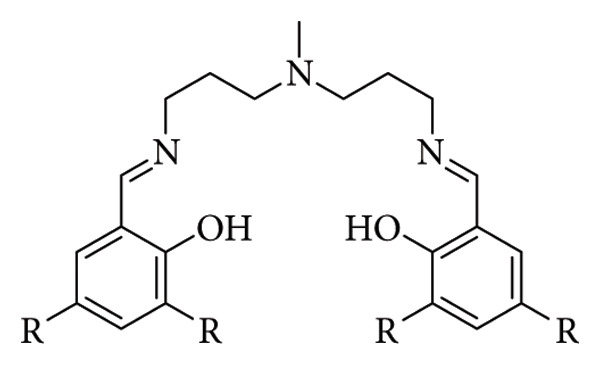
Halogenated Schiff base ligands synthesized by Xu et al. (R = Cl or Br) [84].

**Figure 30:**
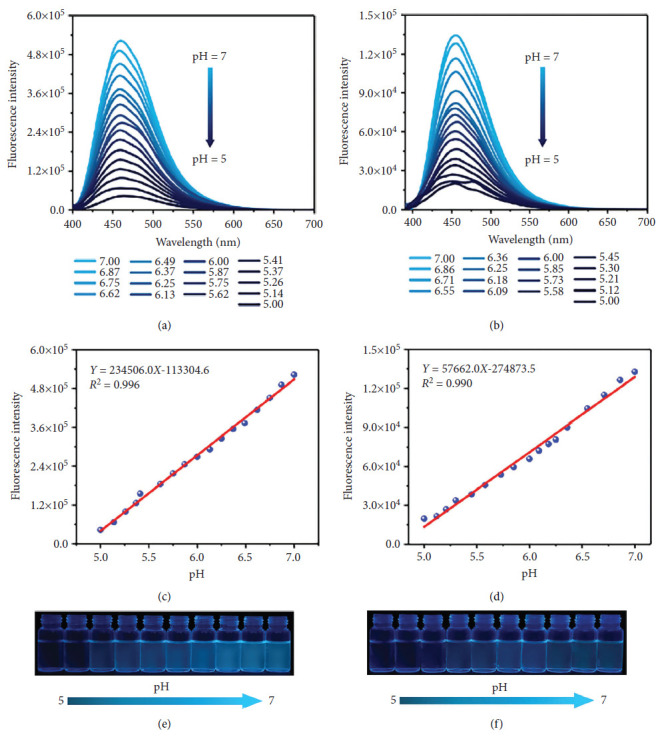
Emission spectra of (a) Zn-Cl-Xu ligand and (b) Zn-Br-Xu ligand at different pH values (*λ*_ex_ = 380 nm and *λ*_ex_ = 370 nm, respectively). The linear relationship between the fluorescence intensity and pH values (5.0–7.0) of (c) Zn-Cl-Xu ligand and (d) Zn-Br-Xu ligand. Fluorescence photos of (e) Zn-Cl-Xu ligand and (f) Zn-Br-Xu ligand at different pH values (5.0–7.0) under 365 nm UV light [84].

Xu et al. developed two high-performance fluorescence pH sensors based on zinc(II) complexes with halogenated Schiff ligands (Figure 29). Based on structural, optical, and theoretical analyses, the incorporation of halogen atoms was found not only to modulate the surface charge distribution of the benzene ring, enhance molecular conjugation, and strengthen intermolecular interactions but also to introduce additional nonradiative pathways. Furthermore, halogen substitution altered the packing arrangement of the probes, thereby increasing the interaction energy between adjacent molecules and promoting a higher degree of molecular aggregation. Fluorescence measurements across varying pH conditions revealed that between pH 2.0 and 5.0, the fluorescence intensity remained largely unchanged, overall exhibiting quenching behavior. Conversely, in the alkaline range from pH 7.0 to 12.0, fluorescence emission was enhanced, though without significant fluctuations. Remarkably, within the narrow pH window of 5.0–7.0, both halogenated Schiff-base complexes displayed pronounced fluorescence enhancement. The fluorescence intensities of the chlorinated and brominated ligands increased by factors of 12.2 and 6.7, respectively, with both demonstrating excellent linearity within this range (Figure 30) [84].

*Metal ion sensors*. Some Schiff bases can form complexes with specific metal ions. When these complexes form, they can give rise to changes in the fluorescence intensity of the Schiff compound. Developing fluorescent sensors for metal ions is crucial due to their ability to detect these ions sensitively and selectively, which play fundamental roles in biological, chemical, and environmental processes. These sensors are vital in medical and biological applications, such as monitoring cellular health and diagnosing diseases, as well as in controlling environmental pollution and ensuring food safety by detecting hazardous metal contaminants. Furthermore, they are essential in industrial applications to guarantee product quality and monitor manufacturing processes, offering a versatile and accurate tool for a wide range of applications spanning human health, environmental protection, and industrial safety [85].

**Figure 31:**
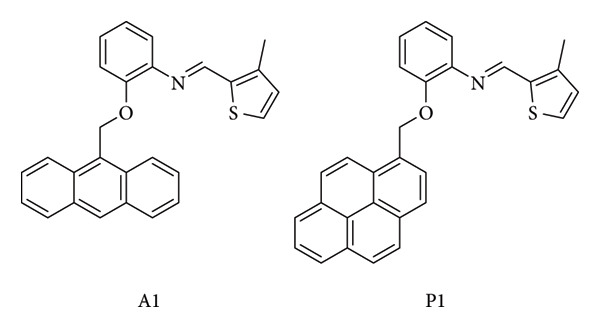
Chemical structures of pyrene-(A1) and anthracene-(P1)-based Schiff bases synthesized by Tümay and Yesilot [86].

Tümay and Yeşilot designed two Schiff base derivatives, A1 (anthracene-based) and P1 (pyrene-based) (Figure 31), intended for the detection of mercury ions (Hg(II)) in food and environmental samples. Mercury contamination is considered one of the most critical global environmental threats, largely due to industrial activities such as mining, wastewater irrigation, and the widespread use of mercury-containing products. Exposure to Hg(II) can result in severe health issues—including organ damage, neurological disorders like Minamata disease, cognitive decline, and even death—owing to its strong affinity for biomolecules and its ability to disrupt enzymatic and protein functions. Even trace levels of Hg(II) in soil or water are highly hazardous, given its strong bioaccumulation factor in the food chain, reaching values as high as 10^6^. The sensors A1 and P1 exhibited detection limits of 12.22 nM and 8.32 nM, respectively, both below the thresholds established by the EPA and World Health Organization (WHO). Their “turn-on” fluorescence response was attributed to 1:1 complex formation with Hg(II) and inhibition of photoinduced electron transfer (PET). Validation by ICP-MS confirmed their reliability, and both sensors were successfully applied to real samples, including rice, black tea, soil, tap water, seawater, and industrial wastewater. The recovery values ranged between 95.68% and 102.52%, showing strong agreement with spiked concentrations. Statistical analysis further indicated no significant differences between the fluorometric method and ICP-MS. To expand their practical utility, P1 was also tested in paper-strip format. Due to the well-documented excimer emission of pyrene groups, which is enhanced in the solid state by π–π interactions, P1 produced a noticeable color change from dark blue to blue-green under 365 nm UV light. This demonstrates its potential as a simple, low-cost test kit for visual Hg^2+^ detection in aqueous solutions. Overall, A1 and P1 proved to be effective, sensitive, and affordable sensing tools for mercury monitoring in food and environmental systems [86].

**Figure 32:**
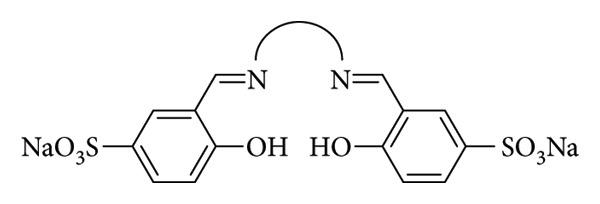
Structures of Schiff bases reported by Reimann et al. [89], featuring bridges between the two nitrogen atoms composed of either a 1,2-benzene ring or a (CH_2_)_n_ spacer (*n* = 2, 3, or 4) [87].

Reimann et al. investigated a series of water-soluble sulfonyl Schiff-base ligands designed to function as fluorescent and colorimetric sensors for metal ions (Figure 32). The research group's focus was on developing sensors for potential biological applications, particularly targeting metal ions that serve as cofactors, metabolic regulators, and oxygen transporters in the human body. The synthesized Schiff base demonstrated selectivity toward metal ions Cu(II), Ni(II), Cr(III), and Co(II) through fluorescence quenching accompanied by colorimetric changes. Notably, the spectroscopic behavior remained unaffected by external factors such as pH, the presence of counterions (F^–^, Cl^–^, I^–^, NO_2_^–^, and NO_3_^–^), or ionic strength (NaCl, Na_2_SO_4_, MgCl_2_, and MgSO_4_). The ability to detect and selectively identify trace amounts of metal ions is critical for drinking water safety and biological studies. The Reimann group evaluated the sensor applicability in water beverages and found that, for copper, the detection limit was lower than the maximum contaminant level established by the EPA for drinking water [87].

**Figure 33:**
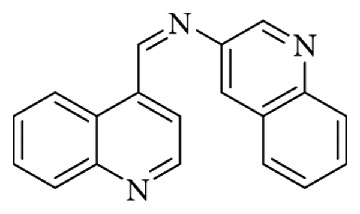
Balasubramanian et al.'s NQQM Schiff base [88].

Balasubramanian et al. published a Schiff base with N-(quinoline-3-yl)-1-(quinoline-4-yl) methenamine (NQQM) containing quinoline constituents (Figure 33). The quinoline group is crucial in the design of fluorescent sensors due to its unique photophysical properties that enable fluorescence in molecules containing it. These molecules are sensitive to environmental changes such as pH, the presence of metal ions, and oxygen concentration, making them ideal for detecting and quantifying these changes. Furthermore, their capacity for structural modification allows for the design of highly selective sensors for specific analytes, finding vital applications in biological, chemical, and medical fields, where they are used for contaminant detection, cellular monitoring, and biological sample analysis. Balasubramanian group focused on the issue of environmental pollution arising from industrial activities, particularly the release of heavy metals such as stannate ions. These ions are widely applied in agriculture (pesticides), the paint and plastics industries, and in various biochemical processes. Interestingly, tin (Sn) itself plays dual roles: while excessive exposure can be toxic, at trace levels it contributes to vital biological functions. It acts as a cofactor in nucleic acid and protein synthesis, supports muscle development, promotes hair growth, maintains body homeostasis, and has been linked to the suppression of cancer cell proliferation. Conversely, tin deficiency has been associated with adverse health effects, including severe respiratory issues, auditory damage, and impaired hemoglobin synthesis. However, the widespread use of Sn(II) compounds is also the primary source of their toxic effects. In their investigation, Balasubramanian and coworkers explored the fluorescence response of the Schiff base ligand NQQM in the presence of various metal ions in methanol. Remarkably, NQQM displayed strong fluorescence enhancement at 461 nm when exposed to Sn(II), attributed to 1:1 complex formation. Under optimized conditions, the limit of detection (LOD) was determined to be 0.026 μM. For comparison, the WHO establishes permissible Sn(II) concentrations ranging from 840 to 8400 μM in bottled water and 2.105 μM in preserved foods. Furthermore, fluorescence intensity was observed to increase proportionally with Sn(II) concentration, confirming the sensitivity of the system [88].

**Figure 34:**
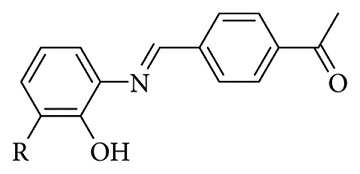
Pang et al.'s Schiff bases (probe S, R = H; probe L, R= OMe) [89].

**Figure 35:**
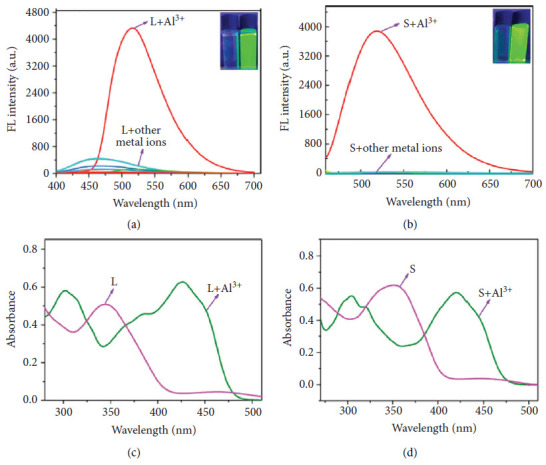
Fluorescence spectra of probes L (a) and S (b) (5.0 × 10^−5^ mol/L) with various metal ions (Al(III), Mg(II), Pb(II), Zn(II), Cu(II), Mn(II), Co(II), Cr(III), Hg(II), Cd(II), Ni(II), and Fe(III), 2.5 × 10^−4^ mol/L) in DMSO/H_2_O solution. UV spectrum of L (c) and S (d) solutions after adding Al(II) ions [89].

Pang et al. have recently introduced two novel Schiff base fluorescent probes (L and S, Figure 34) engineered specifically for the selective detection of Al(III) ions in aqueous solutions. Aluminum is one of the most abundant metals in the Earth's crust and is widely employed across multiple sectors such as food processing, water treatment, pharmaceuticals, environmental technologies, construction, medicine, and the automotive industry. However, its extensive use can increase the levels of aluminum ions (Al(III)) in soils, aquatic systems, and even the atmosphere, posing considerable environmental concerns. The excessive accumulation of aluminum ions in the human body poses severe health risks, potentially contributing to the onset of debilitating conditions such as Alzheimer's, Parkinson's, Menkes, and Wilson's diseases. Furthermore, the WHO established the permissible concentration of Al(III) ions in drinking water at 7.41 mM; the two probes reported by Pang have both high selectivity and sensitivity toward Al(III) ions in aqueous medium: the LOD for Al(III) by L and S was 0.019 8 and 0.0479 mM, respectively, which was much lower than most previously reported probes (Figure 35). Additionally, filter paper strip experiments were performed to establish another potential application of the probes. For probe L/S, the color of the strips was observed to change from ginger/yellow to bright yellow-green under a UV chamber by the naked eye. These observations clearly indicated that the probe L/S immobilized test strips can also be used for monitoring Al(III) in a simple and effective way [89].

*Other important analytes*. Fluorescent sensors based on Schiff bases represent a fundamental tool in detecting various substances in biomedical and environmental applications. The ability of these sensors to detect water, biological molecules, and anions with high sensitivity and selectivity makes them indispensable tools in monitoring water quality, disease diagnosis, and biochemical research. Their modular design and ability to form complexes with different analytes offer unique versatility, allowing adaptation to a wide range of environments and applications. The combination of optical, chemical, and structural properties of Schiff bases provides a robust and effective platform for the development of highly sensitive and specific fluorescent sensors, thus contributing to the continuous advancement in the field of molecular detection and analysis.

**Figure 36:**
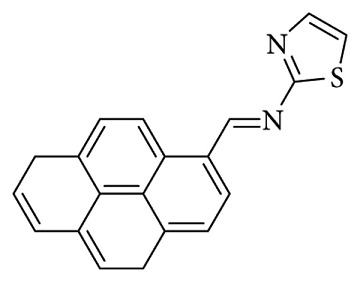
Dash et al.'s pyrene-based Schiff base chemosensor [90].

Dash et al. reported a pyrene-based Schiff base chemosensor (Figure 36) for water detection. Water is not only a fundamental necessity for life itself but also a ubiquitous substance in the field of chemistry. Its significance lies in its dual nature. On the one hand, water serves as the most prevalent yet potentially detrimental impurity, capable of significantly impacting chemical production processes and the research and development of pharmaceuticals. On the other hand, the contamination of industrial oils and chemical fuels with water often results in severe mechanical issues, such as equipment corrosion. Recognizing water's essential role in both our lives and various industrial processes underscores the importance of developing sensors capable of accurately detecting its presence and purity levels. The fluorescent probes synthesized by Dash in DMSO exhibited significant fluorescence enhancement upon the introduction of water. This water-induced emission enhancement was attributed to the synergistic effect of the AIE phenomenon and the suppression of the PET process. The probe enabled the detection of water in DMSO at concentrations as low as 0.50 wt%, with a quantification limit of 1.52 wt%. The analytical applicability of the developed sensor was further demonstrated by successfully detecting moisture in various raw food products, including salt, sugar, and wheat, as well as in commercial products such as detergents [90].

**Figure 37:**
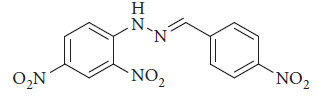
Behura et al.'s water sensor [91].

**Figure 38:**
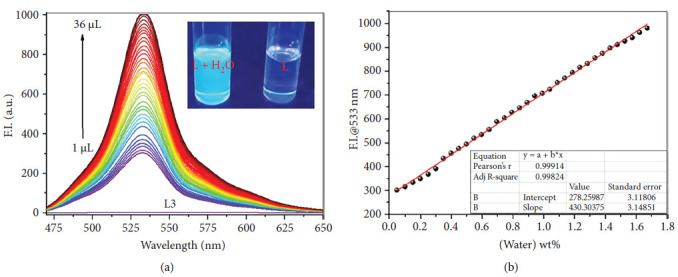
(a) Fluorescence spectral changes of Behura ligand (L3) (10 μM, DMSO) upon incremental addition of water from 1 to 36 μL (inset showed the fluorescent color change after titrating with water; the vials are irradiated with UV-lamp at 365 nm). (b) Calibration plot (fluorescence intensity at 533 nm with increasing water wt%) [91].

Behura et al. were also working on fluorescent sensors of water. This group reported a 2,4-dinitrophenylhydrazine–derived Schiff base (Figure 37) whose water addition into its DMSO solution resulted in a significant fluorescence enhancement at 533 nm with a LOD of 0.002 wt%, which allows the detection of trace amounts (Figure 38). The fluorescence turn-on response occurred due to the formation of aggregates of Schiff base ligand; also, other possible mechanism for trace water detection was attributed to the specific water-ligand interaction and partially to the increase in polarity of the solvent caused by an increase in water concentration. Additionally, the probe showed naked-eye detectable fluorescent color change [91].

**Figure 39:**
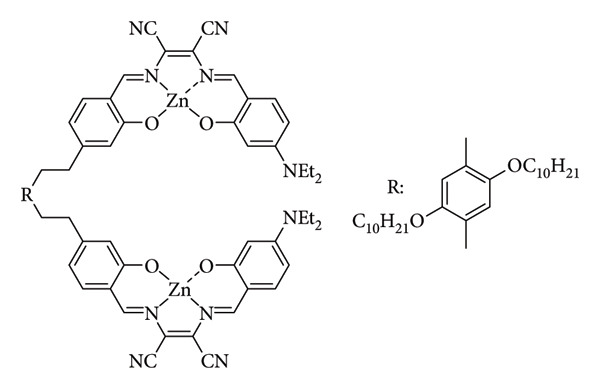
Structure of Munzi et al.'s dinuclear Schiff base complex BA probe [92].

On the other hand, Munzi et al. developed a novel chromophore/fluorophore probe for biogenic amine (BA) detection. BAs are commonly produced during the spoilage of foods such as meat, fish, and seafood. Compounds like cadaverine and putrescine, which are associated with unpleasant odors during meat decomposition, serve as key biomarkers of meat deterioration. Beyond their role as indicators of food quality, BAs exert adverse effects on human health and physiological processes. Elevated concentrations of BAs in spoiled foods are particularly harmful to the central nervous system. Moreover, their interaction with nitrites can lead to the formation of nitrosamines, which are recognized carcinogens. Given the toxic nature of BAs, the Munzi group reported a novel dinuclear Zn(II) Schiff-base complex exhibiting colorimetric and fluorometric responses (Figure 39). The detection mechanism, characterized by high selectivity and sensitivity, involved the formation of stable adducts between the dinuclear complex—acting as a Lewis acidic molecular tweezer—and biogenic di- or polyamines. Competitive assays demonstrated the system's selectivity for BAs, even in the presence of aliphatic monoamines (primary, secondary, or tertiary), heterocyclic amines, and amino acids. Moreover, histamine quantification in fish matrices was successfully achieved through a standard extraction procedure followed by simple colorimetric or fluorometric measurements [92].

**Figure 40:**
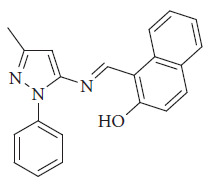
Structure of Krishnan Schiff base BF_3_ probe [93].

**Figure 41:**
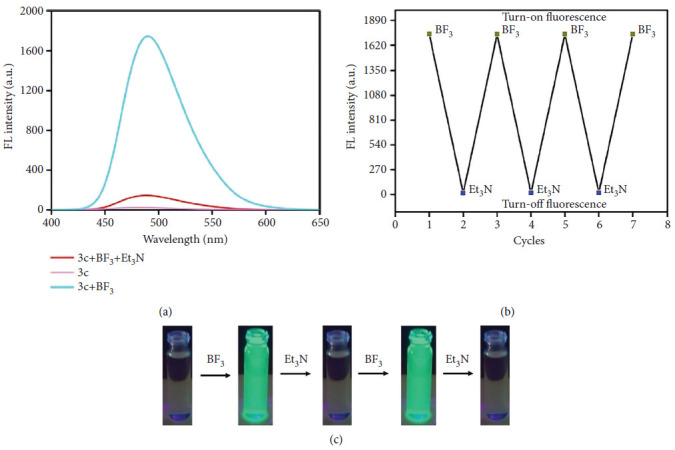
(a) Fluorescence spectra of 3c, 3c + BF_3_, and 3c + BF_3_ + Et_3_N. (b) Emission on cyclic addition of 3c with BF_3_ followed by Et_3_N. (c) Fluorescence color image of 3c with BF_3_ followed by Et_3_N. Krishnan ligand in this figure is named as 3c [93].

Krishnan et al. introduced a Schiff base fluorescent probe (Figure 40) designed for the selective detection of boron trifluoride (BF_3_) in living cells and environmental water samples. BF_3_ assumes a pivotal role in organic synthesis reactions, facilitating isomerization, condensation, and ionic polymerization as a catalyst. Despite its catalytic utility, BF_3_ poses significant hazards due to its highly toxic and corrosive nature. Even at low concentrations, BF_3_ can cause substantial environmental pollution and biological harm. Its exceptional reactivity with metals and organic compounds amplifies its risks. Upon contact with water, BF_3_ can trigger violent explosions, yielding hydrofluoric acid (HF), which poses severe health risks including skin, eye, nasal, and respiratory irritation. Consequently, BF_3_ is classified as a hazardous gas, leading to its prohibition in numerous countries. Krishnan group reported the design of a Schiff base incorporating pyrazole and naphthalene units, developed as both a colorimetric and turn-on fluorescent sensor for rapid detection. The probe demonstrated an exceptionally low detection limit (LOD) of 2.46 nM, significantly lower than values described in earlier studies. Its sensing behavior toward BF_3_ was attributed to an ICT mechanism. Furthermore, the response was shown to be reversible upon the addition of triethylamine (Net_3_), enabling the Schiff base to operate in a controllable “ON–OFF–ON” switching mode (Figure 41). Importantly, the probe was successfully employed for the quantitative detection of BF_3_ in various real water samples, on low-cost paper test strips, and even in living cells, confirming its versatility and practical applicability [93].

**Figure 42:**
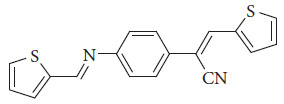
Structure of Huang et al.'s Schiff base hydrazine ion detector [94].

Lastly, the final example we will present is the Huang Schiff base (Figure 42), which was an effective “turn-on” detector of hydrazine ion. Hydrazine and its cation were important but hazardous reagents, which had been widely applied in industry, agriculture, explosives, etc. Hydrazine ion was used as the reducing agent to eliminate solved oxygen in boiler waters and hot-water systems. Hydrazine is recognized as a highly toxic compound capable of inducing severe health problems, including mutagenic effects, skin corrosion, and even cancer. The research group led by Huang introduced the first fluorescent probe for hydrazine ions, designed from a thiophene–cyanodistyrene Schiff base. In aqueous solution, this sensor showed only weak emission within the 450–550 nm range, attributed to the strong PET process that hindered AIE. Upon interaction, the probe demonstrated remarkable selectivity toward both Hg(II) and N_2_H_6_^2+^, producing a distinct “turn-on” fluorescence response through PET inhibition. However, the presence of Cl^−^ ions suppressed the response to Hg(II), enabling highly selective recognition of N_2_H_6_^2+^ alone. The detection limit achieved for N_2_H_6_^2+^ was 1.05 × 10^−7^ M. Furthermore, sensing tests carried out in tap water and samples from the Minjiang River confirmed the suitability of this probe for detecting N_2_H_6_^2+^in complex real-world aqueous environments [94].

## 7. Photovoltaic Materials

Organic solar cells (OSCs) work based on the principle of photovoltaic effect, where light energy is converted into electrical energy. An OSC's active layer comprises a p-type organic semiconductor (donor) and an n-type organic semiconductor (acceptor). When light irradiates the active layer of an OSC, photons are absorbed by the donor and acceptor materials, leading to the formation of excitons, which are bound electron-hole pairs [95]. The excitons then undergo charge separation at the donor-acceptor interface, where the electron is transferred from the donor to the acceptor, creating a separated electron-hole pair. The separated charges, i.e., the electron and hole, are then transported through their respective pathways in the device. The electrons move through the acceptor material toward the cathode, while the holes move through the donor material toward the anode. This movement of charges creates an imbalance of positive and negative charges, generating an electric current. The generated current can be collected and utilized as electrical energy. The efficiency of an OSC is determined by factors such as the absorption of light by the donor and acceptor materials, the efficiency of charge separation and transport, and the prevention of charge recombination [96].

Reducing the cost of non-fused-ring small molecule acceptors in OSCs can be achieved through several strategies:

1. Simplified synthetic routes: Developing simplified and efficient synthetic routes to produce small molecule acceptors can help reduce manufacturing costs. For example, Li et al. reported simplified synthetic routes for low-cost and high-performance non-fused-ring small molecule acceptors [97].
2. Scalable synthesis: Designing small molecule acceptors that can be synthesized on a large scale using cost-effective and readily available starting materials can contribute to cost reduction. This can be achieved by optimizing the synthetic procedures and using commercially available reagents [97].
3. Material engineering: Exploring new materials and molecular structures that exhibit high performance while being cost-effective is another approach. For instance, modifying the molecular structure of acceptor materials to improve their efficiency and reduce their cost has been demonstrated by several studies [98, 99].
4. Process optimization: Optimizing the fabrication processes of OSCs can help reduce costs. This includes developing scalable and efficient deposition techniques, such as solution processing or printing methods, to minimize material waste and increase production throughput [100].

These strategies aim to enhance the cost-effectiveness of non-fused-ring small molecule acceptors, making them more viable for large-scale production and commercialization of OSCs.

*Inorganic solar cells and OSCs*. Inorganic solar cells are typically made from silicon or other inorganic compounds. They are the most common type of solar cells used in commercial applications due to their high efficiency and durability [101]. Three main types of inorganic solar cells exist: monocrystalline, polycrystalline, and thin film. Monocrystalline solar cells are made from a single crystal structure of silicon. They have the highest efficiency rates because they are cut from a single crystal. Polycrystalline solar cells are made from a block of silicon with multiple crystals. These solar cells are less efficient than monocrystalline solar cells but are less expensive. Thin-film solar cells deposit one or more thin layers of photovoltaic material onto a substrate. These types of solar cells are cheaper to produce, but they also have lower efficiency rates [102].

Inorganic solar cells also include other types such as cadmium telluride (CdTe), copper indium gallium selenide (CIGS), and perovskite solar cells (PSCs). These types of solar cells are less common and are still being developed to improve their efficiency and stability.

OSCs have several advantages. They are easy to synthesize and are available at a low cost. They have a high degree of tunability and a high absorption coefficient. Additionally, they are flexible, making them adaptable to various surfaces and applications. However, OSCs also have some disadvantages. They have a high exciton binding energy and low carrier diffusion [103]. The exciton has a shorter diffusion length and should dissociate before recombination. OSCs are mainly synthesized at the lab scale; hence, their properties can be tuned easily, but this also means they may not be as durable or reliable as other types of solar cells [104].

The efficiency of a solar cell measures of how much of the sunlight's energy that hits the cell can be converted into electrical energy. Various factors determine the efficiency of a solar cell, including the materials used, the architecture of the cell, and the manufacturing process. Currently, the highest efficiencies reported for different types of solar cells are as follows: Crystalline silicon solar cells: The highest efficiency reported for a single-junction crystalline silicon solar cell is 26.7%. Thin-film solar cells: For CIGS thin-film solar cells, the highest reported efficiency is 22.6%. OSCs: The highest efficiency reported for an OSC is 16.27%. Amorphous silicon solar cells: The highest efficiency reported for an amorphous silicon solar cell is around 10.5%. Quantum dot solar cells: The highest efficiency reported for a quantum dot solar cell is over 8% [105].

**Figure 43:**
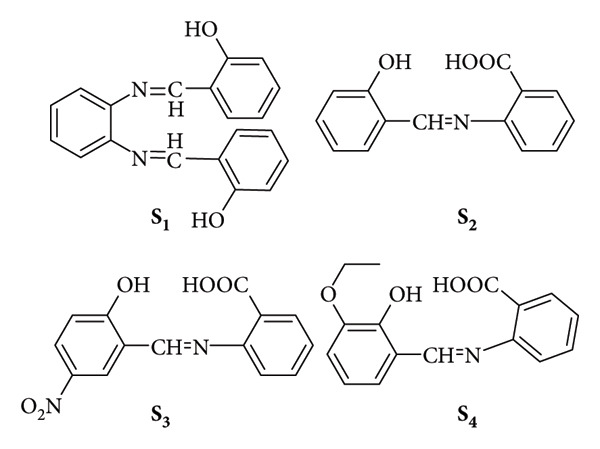
Chemical Structures of the Schiff bases S_1-4_ [107].

*Schiff bases in solar cell applications*. Aromatic Schiff bases are Schiff bases formed from aromatic amines and aldehydes or ketones. They often exhibit interesting electronic and optical properties and have been extensively studied for their potential applications in materials science and optoelectronics [102]. In this review, we will address aromatic Schiff bases due to their optoelectronic properties; numerous works have been reported that focus on dye-sensitized solar cells (DSSCs). This type of technology is based on thin films that are easy to prepare and produce. The efficiency of solar cells based on this technology reaches 12% efficiency using Ru(II). The active part of the Schiff base is the dye, which is responsible for the maximum absorption of UV-vis and near-infrared. Other characteristics of dye are high luminescence. Among the limitations of this technology are its intrinsic material stability (thermal) and oxidation due to the type of electrolyte. To increase the efficiency of this type of design, it is crucial to decrease the oxidation of the material, which generates a higher recombination of the electron-hole pair. Direct contact of the electrolyte with the container glass (ITO or FTO). Inserting chromophores with higher phosphorescence or luminescence in a molecular architecture [106]. To improve the performance of these solar cells, numerous works have been carried out in the last years, some of which are the following. Abdel-Shakour et al. reported synthesis of four Schiff bases (S _1-4_) based on salicylaldehyde (Figure 43). The strong acceptor group (NO_2_) presence in S_3_ is crucial for its superior performance. S_3_ is based on a salicylaldehyde moiety and features a phenyl ring as a donor scaffold. It is connected to a carboxylic group (COOH) that serves as an acceptor and anchoring unit. Notably, S_3_ contains a nitro group (NO_2_), which plays a significant role in its electronic properties and performance in DSSCs. The maximum efficiency of this material is 8.79% [107].

**Figure 44:**
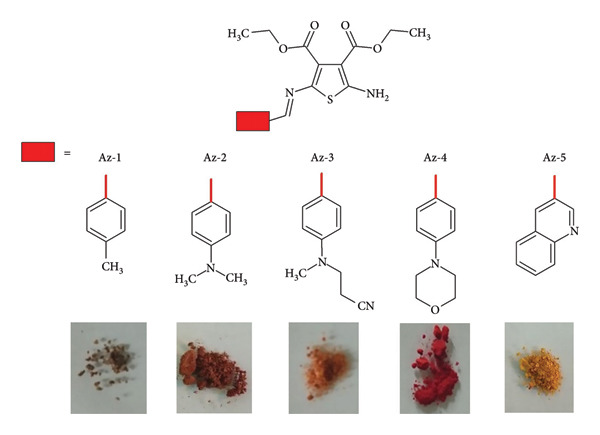
Chemical structures of thiophene imines and their photographs under daylight [108].

Pająk et al. reported the synthesis of five novel unsymmetric thiophene imines, end-capped with an electron-donating amine (−NH_2_) group, via a straightforward melt-condensation reaction of 2,5-diaminothiophene-3,4-dicarboxylic acid diethyl ester with aldehydes. The reactions proceeded with low efficiencies, yielding below 1.3% (Figure 44) [108].

**Figure 45:**
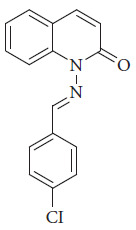
Ligand (L) (1-((4-chlorobenzylidene)amino)quinolin-2(1H)-one) [109].

Salman et al. Schiff synthesized base ligand (L) by condensation reaction of N-amino quinoline-2-one with 4-chlorobenzaldehyde; the results confirmed an octahedral geometry of chrome(III) ion; this material showed good optical properties, and the solar cells have a 7.44% of efficiency (Figure 45) [109].

**Figure 46:**
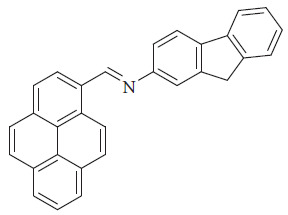
Ligand PyFA [110].

A pyrene-based Schiff base, PyFA, featuring a donor–π–acceptor (D-π-A) architecture, was synthesized and characterized. Photoluminescence analyses confirmed ICT within PyFA, accompanied by green fluorescence emission, while thin-film studies revealed its n-type semiconducting behavior. The device constructed with the configuration ITO | PEDOT:PSS | PyFA | Alq_3_ | Al demonstrated the potential of PyFA as an active material for solar cell applications (Figure 46) [110].

**Figure 47:**
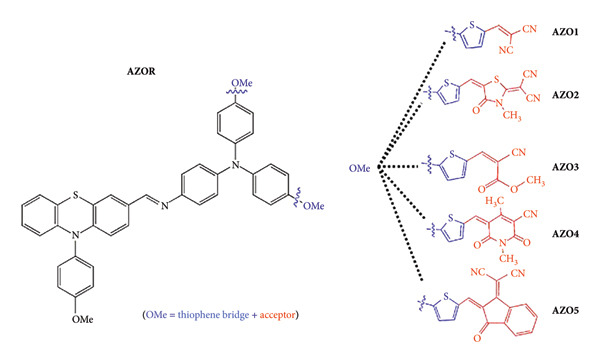
Schematic strategy for designing AZO1, AZO2, AZO3, AZO4, and AZO5 molecules [111].

Zahid et al. designed hole-transporting materials (HTMs) using Schiff bases by molecularly modifying phenothiazine. The configuration was D-π-A and 14%–27% efficiencies were achieved (Figure 47) [111].

**Figure 48:**
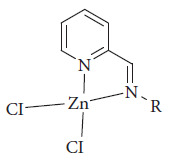
Structure complex with Zn(II) based on pyridine-2-carboxaldehyde [112].

Muddassir et al. synthesized complexes with Zn(II) based on pyridine-2-carboxaldehyde. These compounds have been used as photosensitizers in DSSCs, obtaining an efficiency of 6.52% (Figure 48) [112].

In the last 2 years, there has been an increase in the number of publications based on Schiff base materials due to their good efficiency. Many researchers have become interested due to their easy processing of thin films in smaller quantities of material. For example, Wang et al. used copper acetylacetonate (Cu(acac)_2_) as a passivator in PSCs. The principal objective is to minimize interfacial nonradiative recombination. The formation of Schiff base complexes resulted from the reaction between acetylacetonate and the perovskite, and the efficiency was 24% for this work [113].

**Figure 49:**
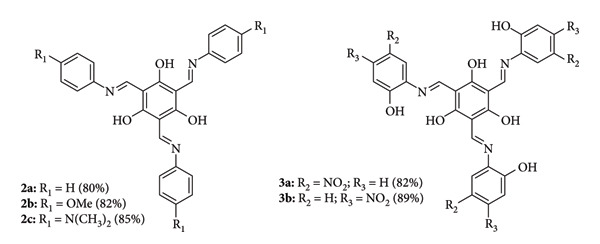
Structure of phloroglucinol aryl-Schiff bases 2a-2c and 3a-3b [114].

Vazquez-Mozencahuatzi et al. synthesized aryl-Schiff base substituted with the methylalcoxy (2b) was used as an electron donor material in photovoltaic devices in configuration: ITO/PEDOT: PSS/2b: PC61BM/Fields metal the performance was inferior. The results showed that the OSCs using 2b as electron donor photogenerated with a power conversion efficiency (PCE) ∼0.4%, which is related to film morphology (Figure 49) [114].

Zheng et al. presented significant findings regarding using Schiff-base copper complexes as HTMs in PSCs made from CsPbBr_3_. The complexes facilitated exciton generation and improved charge transfer from the perovskite layer to the electron transport layer (ETL), thereby increasing the current density. The PCEs achieved were 4.55% for the complexes with R=Cl and 5.71% for those with R=Br [115].

**Figure 50:**
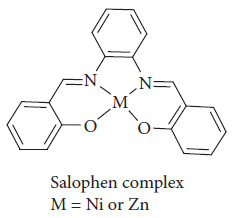
Structure of salophen complexes [116].

Ahmed et al. worked on salophen complexes (Zn(II) and Ni(II)) embedded in PMMA as light concentrators; the coupling of the salophen complexes (Figure 50) with silicon solar cells resulted in a notable increase in photovoltage parameters, and the efficiency of the silicon solar cells was enhanced by approximately 50%, demonstrating the effectiveness of this new approach in improving solar energy conversion [116].

Finally, Karmakar et al. contribute con Azide-bridged manganese(III) complexes with salen-type Schiff base blocking ligands have potential applications in the field of photovoltaics, mainly due to their unique electronic and structural properties; complexes can be engineered to absorb specific wavelengths of light, which is crucial for enhancing the efficiency of photovoltaic devices [117].

**Figure 51:**
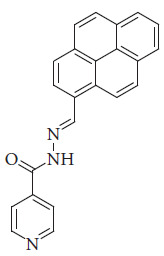
Chemical structure of the PyMls ligand [118].

Tezcan et al. studied a layer of isonicotinohydrazide and pyrene-based Schiff base (PyMIs) (Figure 51) and formed a p-Si semiconductor Al/PyMIs/p-Si/Al Schottky diodes; the diode exhibits photovoltaic behavior with a short-circuit current density (Jsc) of 0.32 mAcm−2 at 100 mW/cm^2^ illumination intensity. These characteristics demonstrate the diode's good rectification behavior and photoconductive properties under varying light conditions [118].

Zahid et al. investigated five newly designed phenothiazine-based HTMs, for high-efficiency PSCs. These materials are engineered using Schiff base chemistry and exhibit improved electronic properties, solubility, and charge transport. Therefore, they facilitate efficient charge generation and separation in PSCs. Schiff bases are emerging as an up-and-coming group of molecules for use in solar cells due to their advantageous structural and electronic properties. These compounds, formed through the reaction between a primary amine and a carbonyl group, stand out for their ability to absorb light over a wide range of the visible spectrum and for their efficient charge transport. Additionally, their ability to form complexes with metals and their chemical versatility make them ideal candidates for solar cells' efficiency and durability. Current research focuses on optimizing these molecules and gaining a deeper understanding of their mechanisms, which will be vital to maximizing their potential in photovoltaic technology. In summary, Schiff bases provide a strong foundation for advancing more efficient and affordable solar cells [119].

## 8. Conclusions

Schiff bases and their metal complexes continue to establish themselves as a versatile platform with impact across multiple areas of contemporary chemistry. This review has integrated findings from the last decade, spanning the development of compounds with therapeutic potential (antimicrobial, antioxidant, and anticancer), their role in sustainable catalysis, luminescent sensors, and emerging applications in photovoltaic devices. Unlike previous reviews focused on isolated aspects, this work provides an integrative overview that connects bioinorganic chemistry with materials science. This transversal approach allows us to identify general trends: (i) metal coordination systematically enhances the bioactivity of Schiff-derived ligands; (ii) the introduction of chiral elements and alternative transition metals broadens the scope of asymmetric catalysis, making it more sustainable; (iii) the rational design of Schiff-based fluorophores opens new opportunities for multipurpose sensing; and (iv) aromatic and metal-containing derivatives are emerging as competitive candidates in next-generation photovoltaic devices. Looking ahead, key challenges include: (a) advancing bioactive compounds into preclinical and clinical stages, (b) improving the stability and recyclability of catalytic complexes, (c) enhancing the sensitivity and selectivity of sensors under real-world conditions, and (d) optimizing the durability of Schiff-based materials in solar and perovskite cells. Addressing these issues will enable a fuller exploitation of these molecules and consolidate their role as a bridge between bioinorganic chemistry and applied materials science.

## Figures and Tables

**Table 1:** Antioxidant assays of some Schiff bases derived from 4-methylaniline and 3-chloro-4-fluoroaniline [44].

Compound	FRAP (μmol TE/G)	DPPH IC_50_	CUPRAC (μmol TE/G)
1a	322 ± 7	6.12 ± 0.04	610 ± 30
1b	407 ± 1	4.91 ± 0.03	1870 ± 20
1c	369 ± 6	5.29 ± 0.06	1190 ± 10
1d	434 ± 5	4.83 ± 0.01	2001 ± 7
1e	448 ± 7	4.61 ± 0.02	2160 ± 20
1f	360 ± 5	5.86 ± 0.03	987 ± 8
1g	489 ± 6	4.45 ± 0.06	2575 ± 5
1h	377 ± 3	5.18 ± 0.01	1430 ± 10
1i	470 ± 8	4.48 ± 0.06	2345 ± 5
2a	2730 ± 10	1.01 ± 0.04	6880 ± 10
2b	4150 ± 10	0.22 ± 0.01	8160 ± 20
2c	4490 ± 20	0.15 ± 0.01	8890 ± 10
2d	3640 ± 20	0.42 ± 0.01	7645 ± 7
2e	3860 ± 10	0.36 ± 0.02	7850 ± 20
2f	4230 ± 10	0.19 ± 0.01	8880 ± 20
2g	3450 ± 10	0.51 ± 0.03	7310 ± 30
2h	3240 ± 20	0.67 ± 0.02	7126 ± 5
2i	4020 ± 10	0.29 ± 0.01	8010 ± 20
Trolox		0.04 ± 0.01	

**Table 2:** IC_50_ values for DPPH assays of copper complexes [45].

Compound	IC_50_ (μM)
AA	0.42
1	7.84
2	9.82
3	3.57

**Table 3:** IC_50_ values of the complexes against four human tumor cell lines: SMMC-7721, MDA-MB-231, and CNE-2Z [51].

Compounds	CNE-2Z	MDA-MB-231	A549	SMMC-7721
Complex 1	20.1 ± 0.7	15.4 ± 0.5	37.7 ± 0.7	55 ± 1
Complex 2	2 ± 2	8.8 ± 0.3	23 ± 3	22 ± 1
Complex 3	17.2 ± 0.1	16.0 ± 0.8	43.3 ± 0.6	73 ± 2
Cisplatin	4.54 ± 0.04	4.02 ± 0.08	15.7 ± 0.5	4.4 ± 0.1

**Table 4:** Percentages of cell inhibition induced by ligands and their metal complexes in the human colon carcinoma cell line [52].

Compound	% of cell inhibition after 72 h	Compound	% of cell inhibition after 72 h
L1	7.4	7	83.22
1	86.1	8	86.39
2	2.3	9	73.3
3	0	HL3	84.4
4	20.7	10	87.5
5	0	11	87.9
6	35.1	12	82.7
HL2	70.57	13	36.4

*Note:* Inhibition rate (%) was calculated at 0.01 mM concentration using MTT assay.

## Data Availability

Data sharing is not applicable to this article as no new data were created in this study.
